# Biomarkers and Cognition Study, Singapore (BIOCIS): Protocol, Study Design, and Preliminary Findings

**DOI:** 10.14283/jpad.2024.89

**Published:** 2024-05-21

**Authors:** Y.J. Leow, J.D.J. Wang, A. Vipin, G.K. Sandhu, S.A. Soo, D. Kumar, A.A. Mohammed, F.Z.B. Zailan, F.P.H.E. Lee, S. Ghildiyal, S.Y. Liew, C. Dang, P. Tanoto, I.Y.Z. Tan, W.F.W. Chong, Nagaendran Kandiah

**Affiliations:** 1 Lee Kong Chian School of Medicine - Imperial College London, Nanyang Technological University, 11 Mandalay Rd, 308232, Singapore, Singapore; 2 Duke-NUS Medical School, National University of Singapore, Singapore, Singapore; 3 The First Affiliated Hospital, Sun Yat-sen University, Guangzhou, China; 4 School of Social Sciences, Nanyang Technological University, Singapore, Singapore

**Keywords:** Southeast Asian, biomarkers, protocol, prevention, dementia, Alzheimer's disease

## Abstract

**Background:**

The focus of medicine is shifting from treatment to preventive care. The expression of biomarkers of dementia and Alzheimer's disease (AD) appear decades before the onset of observable symptoms, and evidence has emerged supporting pharmacological and non-pharmacological interventions to treat modifiable risk factors of dementia. However, there is limited research on the epidemiology, clinical phenotypes, and underlying pathobiology of cognitive diseases in Asian populations.

**Objectives:**

The objectives of the Biomarkers and Cognition Study, Singapore(BIOCIS) are to characterize the underlying pathobiology of Cognitive Impairment through a longitudinal study incorporating fluid biomarker profiles, neuroimaging, neuropsychological and clinical outcomes in a multi-ethnic Southeast Asian population.

**Design, Setting, Participants:**

BIOCIS is a 5-year longitudinal study where participants are assessed annually. 2500 participants aged 30 to 95 will be recruited from the community in Singapore. To investigate how pathology presents with or without minimal clinical symptoms and vice versa, CI and unimpaired individuals will be recruited. Participants will undergo assessments to characterise biomarkers of dementia through neuroimaging, fluid biomarkers, cognitive assessments, behavioural and lifestyle profiles, retinal scans and microbiome indicators.

**Results:**

Since commencement of recruitment in February 2022, 1148 participants have been enrolled, comprising 1012 Chinese, 62 Indian, and 35 Malay individuals. Mean age and education is 61.32 years and 14.34 years respectively with 39.8% males. 47.9 % of the cohort are employed and 32.06% have a family history of dementia. The prevalence of cerebral small vessel disease is 90.2% with a mean modified Fazekas white matter hyperintensity score of 4.1.

**Conclusion:**

The BIOCIS cohort will help identify novel biomarkers, pathological trajectories, epidemiology of dementia, and reversible risk factors in a Southeast Asian population. Completion of BIOCIS longitudinal data could provide insights into risk-stratification of Asians populations, and potentially inform public healthcare and precision medicine for better patient outcomes in the prevention of Alzheimer's disease and dementia.

## Introduction

**T**he focus of medicine is shifting from treatment to prevention. In the field of dementia, we have gained profound insights into early identification of Alzheimer's disease(AD) and other dementias, pharmacological and non-pharmacological treatments for dementia, as well as modifiable risk factors to slow down and prevent cognitive decline. Emerging trials and evidence from anti-amyloid monoclonal antibodies (1), as well as multidomain lifestyle intervention, World Wide FINGERS (2, 3) have highlighted the importance of early identification of dementia, for preventive care and treatment of underlying disease at the earliest stage.

Due to the projected increases in global ageing and population growth, it is estimated that there will be 152.9 million cases of dementia worldwide by 2050 (4). The two main types of dementia are AD and Vascular Dementia(VAD), ranging from 40 to 75% (5) and 20 to 30% (6) of all dementia cases respectively, both which symptoms include progressive impairment in memory and/or other cognitive functions. There is growing awareness on the importance of identifying and utilising biomarkers to better prognosticate and intervene in the realm of preventive medicine. Biomarkers of dementia appear decades before the onset of symptoms (7) and provide a pivotal opportunity period for early diagnosis of cognitive disorders. Pathological changes in the brain, blood, retinal and microbiome can be detected in the prodromal stage of the disease, also known as mild cognitive impairment(MCI), before physical and clinical manifestations of symptoms (8, 9, 10). The prevalence of MCI is estimated to be 15.56% worldwide in community-dwelling adults aged 50 years and older (11), and represents an important at-risk group for characterizing biomarker profiles, disease progression and clinical outcomes of AD and dementia.

There is increasing research on the at-risk population of MCI. However, our understanding of the epidemiology of dementia in Asian populations, particularly within Southeast Asian populations, is limited. With increasing research outcomes in Southeast Asia(SEA) on dementia, the prevalence of AD and VAD in SEA has been reported to be different from Western populations (12, 13). Recent studies have shown that plasma, brain imaging, retinal and microbiome biomarkers are representative of accelerated neurodegeneration which occurs before cognitive decline (14, 15). Therefore, investigating the underlying pathobiology of cognitive impairment among Southeast Asians through characterization of fluid and neuroimaging biomarker profiles, disease progression, neuropsychological and clinical outcomes would be a valuable endeavour.

Despite its small geographical size and young history, Singapore has diverse representations of populations originating from its immigratory history, consisting of Chinese(74.3%), Malays(13.5%), Indians (9%) and Others/Eurasians(3.2%) (16). These three major ethnicities in Singapore are representative of genetic diversity across Asia. With the rise of immigration globally, research on a Singaporean cohort has potential to benefit populations across Asia and worldwide, as there is an underrepresentation of research in Asian populations. Singapore has a strong reputation as the medical and research hub of SEA with the highest physician ratio per 1,000 people (17). Therefore, the Biomarkers and Cognition Study, Singapore(BIOCIS) cohort will aid in the study of cognitive impairment and dementia in SEA - to identify multifactorial factors correlated with higher risk of cognitive decline.

BIOCIS aims to characterize underlying mechanisms of cognitive impairment and dementia by identifying fluid biomarker profiles, neuroimaging and retinal changes, neuropsychological and clinical outcomes in a multiethnic Southeast Asian population. Given recent findings, there is strong evidence to pursue biomarker-based evaluation in cognitively normal individuals as well as those with Subjective Cognitive Decline (SCD) and MCI to understand the trajectory of AD and other types of dementia.

### Dementia and AD Biomarker Profiles in Southeast Asia

#### Blood

Blood-based biomarker characterization analyses have been shown to be comparable to CSF biomarkers in detecting AD and predicting disease progression (18). Amyloid-β peptide(Aβ), tau proteins, plasma proteins, or lipids have shown usefulness in AD diagnosis (19), and there is increasing research on novel biomarkers such as Aβ42/Aβ43 (20) and phosphorylated Tau at position 181(P-tau181). Blood biomarkers such as Neurofilament Light Chain(NfL), Glial fibrillary acidic protein(GFAP), and P-tau181 have been shown in MCI patients to be a predictor for progression to AD (21, 22). Interestingly, blood-based genetic biomarkers have been shown to have an interaction effect with neuroimaging biomarkers. A recent study illustrated that the association between White Matter Hyperintensity(WMH) and grey matter loss is more pronounced in Apolipoprotein E ε4(APOE-ε4) non-carriers than APOE-ε4 carriers in the cognitively unimpaired(CU) and early-stage dementia populations (23). Other recent research similarly indicate a low prevalence of APOE-ε4 and Amyloid-β (24) in Asians compared to western cohorts. European cohorts have shown a APOE-ε4 prevalence of up to 61%, with Asians showing estimates of 41.9% (25). Blood biomarkers could potentially be a less invasive and easily detectable biomarker for AD. Despite these progresses, the contributions and pathobiology of blood biomarkers to disease trajectory and prevalence in Asians requires further definition.

#### Neuroimaging

Neuroimaging can identify underlying causes of dementia through the identification of patterns of atrophy as well as presence and distribution of vascular lesions. Structural imaging studies on brain volumetric changes in dementia have demonstrated associations of dementia with atrophy in the medial temporal lobes, as well as the parietal, frontal and occipital lobes (26). Cerebrovascular disease(CVD) has been increasing shown to be linked to cognitive impairment (27). It has been demonstrated that severity of AD was associated with increased burden of CVD visualized as WMH (28). Functional imaging studies have also shown differences in resting-state connectivity in dementia patients compared to controls (29). Furthermore, there is increasing evidence that WMH is more prevalent in Asia compared to western cohorts and more highly associated with vascular risk factors and poorer cognitive performance (12, 30). Therefore, it would be worthwhile to study brain morphology features in a Southeast Asian cohort and identify neuroimaging risk factors of dementia unique to Asians.

#### Cognitive Assessments, Mood, Behavioural, and Lifestyle Questionnaires

Baseline measures of cognitive function can provide important information about dementia risk years before clinical diagnoses of dementia (31). For example, episodic memory has been widely reported to be first implicated in AD (32), and executive function affected in vascular MCI (33). It has also been shown that subjects with VAD, exhibit impairment in episodic memory, several years before clinical diagnoses, suggesting that circulatory disturbance may affect cognitive performance before the occurrence of stroke that leads to clinical VAD (34). Hence, cognitive profiles observed in individuals who later develop dementia may serve as indicative cognitive biomarkers of dementia and/or its subtypes. Current research indicate that individuals with MCI or subjective memory complaints who do not progress to dementia, perform better at baseline as compared with individuals that progress to dementia on a range of neuropsychological measures (35). Neuropsychological assessments(NPAs) can make important contributions to predicting progression to dementia while individuals are still in early stages of subjective memory complaints or MCI stage. However standard NPAs were mostly developed in homogeneous English-speaking societies(i.e., for non-tonal native language speakers), and growing research suggest that these tests and normative data may not be suitable for culturally different and bilingual populations such as Southeast Asia (36, 37). Therefore, the BIOCIS cohort will serve as a good source of normative data from a bilingual population.

Behavioural changes are one of the earliest observable changes in MCI (38) and dementia (39). In patients with MCI, such changes are common, observed along the severity spectrum of cognitive impairment and known as Neuropsychiatric symptoms(NPS), -“noncognitive behavioural and psychological symptoms of dementia” (40). A Mild Behavioural Impairment Checklist(MBI-C) was developed recently to assess five domains of NPS – Decreased motivation, emotional dysregulation, impulse control, social inappropriateness and abnormal beliefs/perceptions (41). NPS are present in Western and Asian cohorts, and include psychosocial and lifestyle factors such as depression, anxiety, stress and sleep issues (42). However, there are likely to be differences between the two cohorts. For example, apathy may be more difficult to detect and characterize in Asians (43), while Caucasians report appetite changes and apathy more frequently than Chinese (44). There are differences in behavioural profiles as well between dementia subtypes, with depression and apathy more commonly reported in VAD (6). It is therefore essential to characterize behavioural changes of southeast Asians for earlier and more accurate detection of prodromal dementia by clinicians and caregivers.

#### Gut and Microbiome

Altered gut microbiota has been reported in individuals with MCI and dementia (45). Bacteria can communicate and induce changes within the Central Nervous System (CNS) through producing and secreting neurotransmitters in full or as metabolites/fractions, and secreting amino acids and other compounds including short-chain fatty acids and folate, or combinations thereof, which have the ability to communicate and cause change within the CNS. 11 urinary metabolites were identified to be significantly altered in dementia patients, including glucose, guanidinoacetate, urocanate, hippuric acid, cytosine, 2- and 3-hydroxyisovalerate, 2-ketoisovalerate, tryptophan, trimethylamine N oxide, and malonate, suggesting that urine metabolomics may be useful for developing a test capable of diagnosing and distinguishing MCI and dementia from cognitively healthy controls (45). As gut microbiome is influenced by genetics, diet, lifestyle and geographic locations, the BIOCIS Southeast Asian cohort will present a unique community of microorganisms that could be investigated as microbiome biomarkers of dementia.

#### Retinal

Posited as low-cost and non-invasive, retinal imaging is increasing shown to be an useful dementia biomarker tool in dementia research (46). The retina, due to its anatomical and physiological similarities with the brain, can provide new insights to neurodegenerative processes beyond what is understood from neuroimaging (47). The retina shares an embryonic origin with the CNS in the neuroepithelium, and as it is the only CNS tissue that is directly optically accessible, is uniquely suited to highresolution optical imaging such as optical coherence tomography(OCT). The ocular microvasculature, which includes small blood vessels in the eye, has gained attention in recent years for its potential significance in early detection and monitoring of various systemic diseases, including dementia and MCI. Structural changes in the retina have been observed in individuals with MCI as well (48, 49).

Laser Speckle Flowgraphy(LSFG) provides a noninvasive means to study retinal blood flow. LSFG assesses retinal blood flow by analyzing the speckle pattern created when laser light interacts with moving blood cells in the micro vessels of the eye. LSFG provides insights into microcirculation, helping researchers understand how blood flow changes in individuals with dementia. A recent study demonstrated the effectiveness of LSFG in assessing dynamic changes in retinal blood flow, contributing to current understanding of vascular factors in Alzheimer's disease (50). Longitudinal research utilizing LSFG could therefore identify early biomarkers for dementia. By measuring blood flow in the eye over an extended period, researchers can monitor subtle changes that may precede cognitive decline (51). These changes in blood flow patterns, which can reflect alterations in cerebral microcirculation, may offer critical insights into the progression of dementia. The BIOCIS cohort intends to correlate retinal scans with brain, blood and neurocognitive data for novel biomarkers in an Asian setting.

#### Longitudinal Research in Southeast Asians

**Figure 1:**
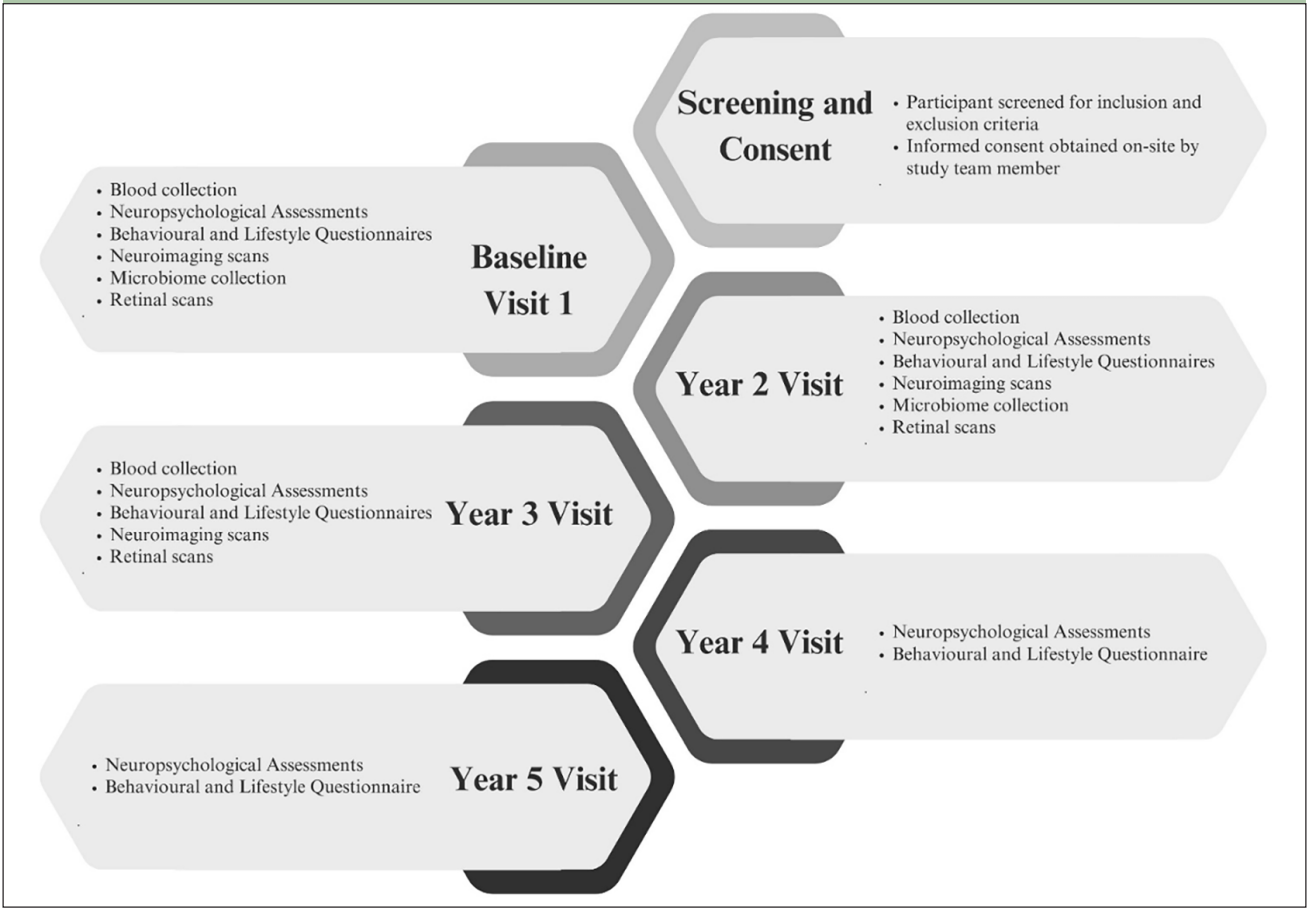
Biomarkers and Cognition Study, Singapore (BIOCIS) Participant Flowchart

Recent studies on the dementia biomarkers have demonstrated that pathology in AD broadly follows a temporal order. Aβ is first to be implicated, followed by tau markers of neuronal injury and brain atrophy. Finally, as the disease pathology progresses, behavioural, memory and other cognitive functions become impaired and symptoms increase (52). However, across all biomarkers and symptomatology, research into community dwelling Southeast Asians is limited (53). There is currently no largescale longitudinal cohort of southeast Asians with neuroimaging, blood biomarkers, cognitive assessments, behavioural and lifestyle profiles, retinal and microbiome markers. Hence, the BIOCIS study will provide comprehensive data that will be vital for understanding the unique aspects and subtypes of dementia within this population, and shape the development of tailored strategies for prevention, early detection and treatment of dementia. Figure 1 presents a summary of BIOCIS study components.

## Methods

### Study design

BIOCIS is a 5-year longitudinal cohort study where research participants are assessed annually. Participants are recruited from the community at Dementia Research Centre(Singapore) (DRCS) at the Lee Kong Chian School of Medicine, Nanyang Technology University(LKC-NTU). Recruitment sources include - DRCS website(www.drcs.sg); public engagement events; family and peer referrals from existing participants, and advertisements. Figure 1 presents the participant flowchart.

### BIOCIS Objectives and Hypotheses

#### Objective 1

To characterize cognitive and behavioural profiles, neurodegenerative, neuroinflammatory, cerebrovascular, metabolic, retinal and fluid biomarkers in CU and cognitively impaired(CI) Southeast Asian subjects.

Hypothesis 1a Neuroimaging and fluid biomarkers of neurodegeneration will predict cognitive deterioration and progression along the dementia spectrum over Timepoints 1 to 5.

Hypothesis 1b Fluid biomarkers of neuroinflammation will correlate with cross-sectional and longitudinal markers of neurodegeneration in predicting cognitive change and progression along the dementia spectrum over Timepoints 1 to 5.

Hypothesis 1c Genetic profiles will influence the cognitive measures, fluid biomarker expression and neuroimaging patterns as well as progression rates to dementia.

#### Objective 2

To study baseline and longitudinal changes in neuroimaging, blood, retinal and microbiome biomarkers in CU and CI Southeast Asian subjects.

Hypothesis 2a Longitudinal changes over Timepoints 1 to 5 in neuroimaging, blood, retinal and microbiome biomarker levels will be correlated to changes in cognition.

Hypothesis 2b APOE genotype will influence the interaction between neurodegenerative, vascular, neuroinflammatory markers on longitudinal cognitive change.

#### Objective 3

To study the structural and functional neuroimaging changes in CU and CI subjects at baseline and longitudinally, together with their interaction with neurodegenerative, cardiovascular, metabolic, neuroinflammatory and genetic markers, with the aim to develop a novel diagnostic MRI toolbox for cognitive disorders among Southeast Asians.

Hypothesis 3a Subjects with elevated Aβ42, Aβ40, P-tau181and NfL will have altered functional connectivity in the default mode network than those with normal plasma amyloid levels.

Hypothesis 3b Subjects with elevated Aβ42, Aβ40, P-tau181 and NfL will have greater grey and white matter changes and altered blood brain barrier permeability than those with normal plasma amyloid-beta and phospho-tau levels.

Hypothesis 3c Subjects with elevated Aβ42, Aβ40, P-tau181 and NfL will have altered brain perfusion compared to those with normal plasma amyloid-beta and phospho-tau levels.

Hypothesis 3d Structural and functional MRI data along with the blood-based biomarkers and neurocognitive measures will allow for the development of novel MRI toolbox for better disease characterisation.

#### Objective 4

To study the correlation between clinical and neuropsychological findings, with blood-based biomarkers in CU and CI subjects at baseline and longitudinally.

Hypothesis 4a Neuropsychological measures in the domains of episodic memory, executive function, attention, processing speed, visuospatial, language will be correlated with biomarkers of neurodegeneration and neuroimaging measures.

Hypothesis 4b Clinical and behavioural measures will be associated with cognitive performance.

Hypothesis 4c Behavioural assessments for depression, anxiety, stress, sleep, MBI will be associated with cognitive performance and progression along the spectrum of dementia.

Hypothesis 4d Subjects with higher AD-typical biomarker levels will demonstrate greater impairment in neuropsychological, clinical and behavioural outcomes.

#### Objective 5

To develop and study the performance of digital biomarkers in the evaluation of cognitive trajectory and behavioural change from longitudinal visits via artificial intelligence in CU and CI subjects.

Hypothesis 5a Using a digital platform to assess cognitive performance and behavioural symptoms will have the capability to identify alterations in cognition and/or behaviour, which will show a correlation with results from conventional pen-and-paper neuropsychological tests.

Hypothesis 5b Digital-based cognitive performance will be correlated with blood-based biomarkers and neuroimaging measures.

#### Objective 6

To characterize associated neuronal pathologies “in a dish” via studies utilizing blood samples from CU and CI subjects, with and without cerebral white matter disease and other neurodegeneration pathologies at baseline and longitudinally.

Hypothesis 6a The blood samples among CI subjects will display evidence of dysregulation of MicroRNAs(miRNAs) particularly in the peripheral blood mononuclear cells (PBMCs), indicating their role in the pathogenesis of neurodegenerative disease.

Hypothesis 6b A regulatory network exists between miRNA, amyloid-beta precursor protein (APP) processing and tau phosphorylation in induced human neuronal cells.

#### Objective 7

To study the association between gut microbiome health and urinary metabolites with fluid biomarkers, cognitive performance, behaviour and neuroimaging in CU and CI subjects at baseline and longitudinally.

Hypothesis 7a Gut microbiota will be associated with greater neuroinflammation resulting in impairment in cognitive function and brain atrophy.

Hypothesis 7b Urine metabolites will correlate with brain atrophy and cognitive performance in subjects with Cognitive Impairment.

#### Objective 8

To investigate retinal biomarkers as possible early indicators for MCI and dementia at baseline.

Hypothesis 8a Retinal biomarkers will exhibit distinct patterns in CI subjects when compared to CU subjects and will be associated with fluid and neuroimaging biomarkers.

#### Objective 9

To study if changes in the retinal-choroidal vessels are correlated to cerebral small vessel disease (CSVD), brain structure and function and amyloid-tau pathology in CI subjects longitudinally.

Hypothesis 9a Changes in the retinal-choroidal vessels will be correlated to presence of CSVD, brain structure function and amyloid-tau pathology in CI subjects.

### Inclusion and Exclusion criteria

Inclusion criteria include - Aged 30 to 95 years; English or Mandarin literacy; intact mental capacity. Mild dementia/vascular-related conditions are allowed if individual has intact mental capacity. Lower age limit of the population is 30 years old, to research early-onset cognitive issues, conduct risk factor analyses including lifestyle, environmental factors and genetics, as well as provide longitudinal insights into cognitive health. Participants with MRI contra-indications will be excluded from MRI scans but allowed to participate in other aspects of the study including neuropsychological evaluations and fluid biomarker studies. Exclusion criteria include - Serious neurological, psychiatric or systemic diseases; history of psychotic disorder, major depressive disorder, alcoholism or drug dependency/abuse within two years.

### Assessments/Outcome measures

#### Demographics, clinical history and physical assessment

Demographics will be collected by self-report. Clinical history of diabetes, hypertension and hyperlipidaemia is defined as - a formal diagnosis from a medical professional, current medication or baseline measures above established cutoffs. Physical assessments include height, weight, walking speed, hand grip strength, blood pressure and heart rate. Lipid profile will be measured using fasting lipids (i.e., total cholesterol, high-density lipoprotein cholesterol, low-density lipoprotein cholesterol, triglycerides) and blood sugar profile will include fasting venous sugar and Glycated hemoglobin(HbA1C).

#### Blood biomarkers

Up to 25ml of blood will be collected from participants at three time-points - Visit 1, 2 and 3. The blood will be used for biochemistry analyses, Single molecule array (SiMOA) analyses, micro-RNA, genetic analyses, and neuroinflammatory panels. Blood biomarker tests include APOE ε2/ε3/ε4, Oaβ, NFL, Aβ42, Aβ40, GFAP, and P-tau181.

#### Neuroimaging

Participants will undergo magnetic resonance imaging(MRI) scan on a 3T Siemens Prisma Fit(Siemens, Erlangen, Germany). Structural MRI will be acquired using T1-weighted MPRAGE sequence. A T2-weighted fluid-attenuated inversion recovery(T2-FLAIR) image will be used to quantify white matter lesions using manual and automatic procedures. DTI will be acquired using a single-shot spin echo echo-planar sequence. Susceptibility-weighted imaging(SWI) and arterial spin labelling(ASL) sequences will also be run. To quantify Staals, Fazekas WMH, medial temporal atrophy(MTA), cerebral microbleeds, lacunes and perivascular spaces(PVS) scores, MRI visual ratings will be performed on T1 and FLAIR MRI sequences by two trained raters blinded to diagnosis according to previously published methods (54, 55). The average MTA rating was used to classify abnormal MTA (56) and Staals scores were assigned to WMH, PVS, microbleed and lacunes ratings based on Staals criteria (57). The T1-weighted MPRAGE sequence was acquired with repetition time of 2000ms, echo time 2.26ms, inversion time 800ms, flip angle 8, 1mm slice thickness, 176 slices, and 1×1×1mm voxel size. The T2-weighted FLAIR Magnetic Resonance Imaging (MRI) sequence was acquired with repetition time of 7000ms, echo time 394ms, inversion time 2100ms, flip angle 120, 1.56mm slice thickness, 192 slices, 0.8×0.8×1mm voxel size. The SWI sequence was acquired with repetition time 28ms, echo time 20ms, flip angle 15, 2mm slice thickness, ROW encoding direction, 72 slices, 0.625×0.625×1mm voxel size. Structural images were pre-processed using FreeSurfer (https://surfer.nmr.mgh.harvard.edu/, software version 7.2). For each participant's T1 images, automated segmentation and cortical parcellation was carried out using the FreeSurfer “recon-all” processing stream, which included motion correction, removal of nonbrain tissue, automated Talairach transformation, intensity correction, volumetric segmentation, and cortical surface reconstruction and parcellation. Task-free fMRI data will be acquired using two runs of T2-weighted echo planar sequence. Subjects are instructed to remain still and keep their eyes open with fixation on the centre cross of the screen. Functional images will be inspected for motion and radio frequency spikes to minimize image artefacts.

#### Cognitive and Neuropsychological Assessments

A formal, standardized battery of neuropsychological tests will be administered to the participant by an experienced research staff, either in English or Mandarin. The battery of tests were selected based on their validity, sensitivity, level of difficulty, reliability and ease of administration. The neuropsychological battery includes the Clinical Dementia Rating(CDR) and tests of: Global cognition - Montreal Cognitive Assessment(MoCA) and Visual Cognitive Assessment Test(VCAT); Learning/Memory - Rey Auditory Verbal Learning Test(RAVLT) Delayed, Logical Memory immediate and delayed, Rey Complex Figure Test(RCFT) Delayed, Free and Cued Selective Reminding test(FCSRT); Executive Function – Trial Making Test B, Test of Practical Judgement(TOP-J), Colour Trials 2, Processing speed – Colour Trials 1, Symbol Digit Modalities Test(SDMT); Language – Semantic fluency(Animals); Visuospatial skills – Block Design, RCFT Copy; Attention – Digit Span(DS) Forward; and Working Memory – DS Backwards. DRCS is developing digital and gamified assessments to conduct concurrent validation tests, improve tests sensitivity and accessibility, and enable remote monitoring.

#### Mood, behavioural and lifestyle questionnaires

The self-reported mood, lifestyle and behavioural questionnaires included: Depression Anxiety Stress Scales(DASS); The Mild Behavioural Impairment-Checklist(MBI-C); Dementia-Quality of Life Instrument(DEM-QOL); International Physical Activity Questionnaire(IPAQ); Fried Phenotype Frailty; Pittsburgh Sleep Quality Index(PSQI); and Subjective Memory Complaints Questionnaire(SMCQ).

### Microbiome sample collection

Stool and urine samples will be collected from participants at Visit 1 and 2. Diet questionnaires will be administered as well, including a Seven-day food diary and Food Frequency Questionnaire.

### Retinal scans

Participants will be seated and imaged with the ultrawide-field wept-source optical coherence tomography angiography(SS-OCTA)(TowardPi Medical Technology Co., Ltd., Beijing, China). The SS-OCT is a third-generation spectral domain OCT with the ability to scan 24× 20mm in a single shot and a larger field of view(extending the imaging field to 70°), allowing for detailed analysis of retinal layers and pathological changes. LSFG offers a complementary perspective by assessing retinal blood flow in real-time. Participants are seated in front of the LSFG device in a dimly lit room to optimize image quality. A small probe is gently placed on the orbit, which emits laser light to capture speckle patterns created by blood flow in the retina.

### Statistical Analyses Plan

Outcome measures will be fluid, brain imaging, neuropsychological, retinal and microbiome data. Baseline characteristics will be computed to summarize demographic and clinical characteristics of the BIOCIS cohort. Univariate and Regression analyses will be conducted to investigate associations between outcome variables and dementia progression. Latent variable modelling techniques including structural equation modelling (SEM) will be utilized to explore relationships among outcome. Multiple regression models will be used to examine the predictive value of different demographics, clinical and outcome variables on cognitive decline over time. Linear mixed models will be employed to analyze the longitudinal changes in outcome variables over the 5-year period. Adjustment for clinical and statistical confounders will be incorporated into all analyses. Statistical analyses will be performed using IBM SPSS Statistics for Windows, Version 29.0. Armonk,NY:IBM Corp and RStudio (2022.12.0 Build 353 © 2009–2022 Posit Software PBC). All statistical tests were two-sided and performed at 5% level of significance.

### Power Calculations

Group sample sizes of 1000 CI and 1000 CU patients will achieve >80% power to detect small effect sizes. The type I error is adjusted for the multiple comparisons within each group of variables being tested using Bonferroni correction approach. Overall significance level is set at 5%. Assuming a 20% participant attrition rate as the worst-case scenario, the target sample size would be 1250 participants per group yielding a total sample size of 2500. Power calculation is performed using a two-sided two-sample equal-variance t-test via PASS software (PASS 2022 Power Analysis and Sample Size Software (2022). NCSS, LLC. Kaysville, Utah, USA).

## Preliminary Data

### Diagnostic Classification of participants

Participants are classified as Healthy Controls(CN), Subjective Cognitive Decline(SCD), MCI and Mild Dementia(AD) according to published criteria (58) and the National Institute on Aging-Alzheimer's Association(NIA-AA) criteria (59): Participants with no subjective memory complaints and unimpaired cognitive scores were classified as CN; Participants with subjective memory complaints and unimpaired cognitive scores were classified as SCD; Participants with subjective memory complaints and had impaired cognitive scores (i.e., 1.5 standard deviations below the CN mean), with no functional deficits will be classified as MCI. Participants with subjective memory complaints and impaired cognitive scores will be classified as dementia. Tables Table 1, Table 2, Table 3 presents the demographics, fluid biomarkers, neuroimaging and cognitive data. Microbiome and retinal data are currently not available in the preliminary data and are therefore excluded from this paper.

**Table 1:** Demographics, vascular and clinical history

	CN n=385	SCD n=239	MCI n=510	Dementia n=14	Total n=1148
Age	58.31 ± 10.75	57.93 ± 9.34	64.68 ± 9.15	79.43 ± 11.18	61.32 ± 10.48
Sex (Male)*	154 (40.1%)	74 (%31)	222 (43.7%)	7 (50%)	457 (39.8%)
Education†	15.16 ± 3.42	15.00 ± 3.03	13.58 ± 3.80	8.79 ± 6.45	14.34 ± 3.69
Ethnicity					
Chinese*	329 (85.7%)	220 (92.1%)	451 (88.8%)	12 (85.7%)	1012 (88.4%)
Malay*	15 (3.9%)	6 (2.5%)	13 (1%)	1 (7.1%)	35 (3.1%)
Indian*	20 (5.2%)	10 (4.2%)	31 (1%)	1 (7.1%)	62 (5.4%)
Eurasian*	4 (1%)	0 (0%)	2 (0%)	0 (0%)	6 (0.5%)
Others*	16 (4.2%)	3 (1.3%)	11 (0%)	0 (%)	30 (2.6%)
Languages spoken†	2.17 ± 0.92	2.26 ± 1.20	2.10 ± 1.02	2.00 ± 0.88	2.16 ± 1.03
Employment(Employed)*	210 (54.8%)	119 (49.8%)	216 (42.5%)	3 (21.4%)	548 (47.9%)
Employment type (Professional)*	215 (56.1%)	137 (57.3%)	265 (52.6%)	4 (28.6%)	621 (54.5%)
Last drawn (<SGD$5,000)*	126 (34.3%)	84 (35.6%)	198 (40.0%)	7 (50.0%)	415 (37.3%)
Housing type (4/5 room apartment)*	132 (34.4%)	69 (29.0%)	173 (33.9%)	7 (50.0%)	380 (33.2%)
Medical history					
Diabetes*	58 (15.1%)	29 (12.1%)	98 (19.2%)	3 (21.4%)	188 (16.4%)
Hypertension*	128 (33.2%)	61(25.5%)	223 (43.7%)	9 (64.3%)	421 (36.7%)
Hyperlipidemia*	278 (72.2%)	174 (72.8%)	395 (77.5%)	9 (64.3%)	856 (74.6%)
Currently Smoking(Yes)*	9 (2.3%)	9 (3.8%)	12 (2.4%)	0 (0.0%)	30(2.6%)
Current Alcohol Consumption(Yes)*	131 (34.2%)	103 (43.1%)	195 (38.5%)	3 (21.4%)	432 (37.8%)
Family history of dementia(1st degree)	116 (30.1%)	94 (39.3%)	161 (31.6%)	3 (21.4%)	375 (32.7%)
Vascular assessments					
BMI†	23.58 ± 3.57	23.44 ± 3.58	24.01 ± 3.86	23.53 ± 5.00	23.74 ± 3.72
Systolic BP†	127.63±19.78	123.02 ± 18.37	129.63 ± 17.16	134.86 ± 17.51	127.64 ± 18.50
Diastolic BP†	77.78 ± 11.49	77.46 ± 11.82	78.39 ± 10.21	77.61 ± 13.73	77.98 ± 11.03
TC†	5.30 ± 1.08	5.40 ± 1.12	5.23 ± 1.08	4.41 ± 1.40	5.28 ± 1.10
HDL-C†	1.66 ± 0.44	1.67 ± 0.41	1.66 ± 0.45	1.52 ± 0.30	1.66 ± 0.44
LDL-C†	3.14 ± 0.94	3.21 ± 0.91	3.02 ± 0.90	2.42 ± 1.18	3.09 ± 0.92
Fasting Glucose†	5.14 ± 1.02	5.12 ± 1.06	5.32 ± 1.12	6.36 ± 3.76	5.22 ± 1.15
HBA1C%†	5.87 ± 0.71	5.82 ± 0.64	5.99 ± 0.67	6.34 ± 2.31	5.92 ± 0.72


Note. † = mean ± Standard Deviation, * = Frequency (percentages); Abbreviations: CN = Cognitively Normal, MCI = Mild Cognitive Impairment, SCD = Subjective Cognitive Decline, BMI = Body Mass Index, BP = Blood pressure, TC = Total cholesterol, HDL-C = High-density lipoprotein cholesterol, LDL-C = low-density lipoprotein cholesterol, HBA1C = Glycated hemoglobin

**Table 2:** Fluid Biomarkers and Neuroimaging Visual Ratings

**Fluid Biomarkers**					
	**CN n=257**	**SCD n=151**	**MCI n=308**	**Dementia n=8**	**Overall n=724**
APOE ε2 gene*,**	45 (15.0%)	19 (10.4%)	39 (11.0%)	0 (0%)	103 (12.2%)
APOE ε3/ε3 genotype**	210 (69.8%)	132 (72.5%)	246 (69.7%)	7 (87.5%)	595 (70.5%)
APOE ε4 gene*,**	46 (15.3%)	31 (17.0%)	68 (19.3%)	1 (12.5%)	146 (17.3%)
Oaβ†	0.53 ± 0.41	0.48 ± 0.28	0.55 ± 0.38	0.69 ± 0.46	0.53 ± 0.37
NfL†	16.31 ± 9.45	14.83 ± 12.04	18.14 ± 7.93	35.70 ± 22.20	17.11 ± 9.42
Aβ42†	5.16 ± 1.56	5.01 ± 1.68	5.32 ± 1.52	5.99 ± 2.43	5.22 ± 1.56
Aβ40†	87.61 ± 24.72	84.68 ± 31.14	96.88 ± 22.32	118.50± 17.68	91.91±25.14
GFAP†	82.14 ± 46.23	69.04 ± 36.17	100.07 ±49.24	201.50±135.06	89.75 ± 49.07
P-tau181	19.72 ± 7.10	19.25 ± 9.25	19.73 ± 6.60	34.10 ± 15.98	19.75 ± 7.33
**Neuroimaging Visual Ratings**					
	**CN n=270**	**SCD n=152**	**MCI n=287**	**Dementia n=6**	**Overall n=715**
Total Fazekas†	3.62 ± 2.70	3.95 ± 2.74	4.49 ± 2.89	10.33 ± 2.34	4.10 ± 2.86
Low Fazekas(0–4)**	190 (70.4%)	94 (61.8%)	162 (56.4%)	0 (0%)	446 (62.4%)
Med Fazekas(5–8)**	64 (23.7%)	49 (32.2%)	95 (33.1%)	1 (16.7%)	209(29.2%)
High Fazekas(9–12)**	16 (5.9%)	9 (5.9%)	30 (10.5%)	5 (83.3%)	60 (8.4%)
Confluent WMH**	12 (4.4%)	6 (3.9%)	22 (7.7%)	4 (66.7%)	44 (6.2%)
Staals†	0.88 ± 0.96	1.04 ± 1.00	1.32 ± 1.11	2.33 ± 1.03	1.10 ± 1.06
MTA†	2.64 ± 1.00	2.62 ± 1.05	2.90 ± 1.10	5.17 ± 1.72	2.76 ± 1.09
MB count†	0.19 ± 0.53	0.18 ± 0.51	0.25 ± 0.69	6.00 ± 13.73	0.26 ± 1.40
Lacunes Total†	0.51 ± 1.19	0.74 ± 2.50	1.03 ± 2.14	4.00 ± 8.85	0.80 ± 2.09
PVS Total†	2.05 ± 0.88	2.28 ± 0.97	2.43 ± 0.95	2.67 ± 1.63	2.26 ± 0.95


Note. *=heterozygote or homozygote, †=mean ± Standard Deviation, **=Frequency (percentages); Abbreviations: CN=Cognitively Normal, MCI=Mild Cognitive Impairment, SCD=Subjective Cognitive Decline, APOE=Apolipoprotein E, Oaβ = Aβ oligomers, NFL=Neurofilament Light Chain, Aβ=Amyloid Peptides, GFAP=Glial fibrillary acidic protein, P-tau181=Phosphorylated Tau at position 181, WMH=White Matter Hyperintensity, MTA=Medial Temporal Atrophy, MB=Cerebral Microbleeds, PVS=Perivascular Spaces

**Table 3:** Neuropsychological, Mood, Behavioural and Lifestyle Assessments/Questionnaires

	CN n=385	SCD n=239	MCI n=510	Dementia n=14	Overall n=1148
**Neuropsychological Assessments**					
**Global cognition**					
CDR 0	385 (100%)	115 (48.1%)	203 (39.8%)	0 (0%)	703 (61.2%)
CDR 0.5	0 (0%)	124 (51.9%)	306 (60%)	0 (0%)	430 (37.5%)
^MoCA	27.7 ± 1.33	25.9 ± 2.39	23.8 ± 3.20	13.2 ± 7.58	25.4 ± 3.41
^VCAT	27.4 ± 2.36	27.7 ± 2.08	25.5 ± 3.62	14.5 ± 8.56	26.4 ± 3.50
Learning/Memory					
^RAVLT Immediate	53.01 ± 8.96	52.07 ± 9.06	43.60 ± 11.09	33.00 ± 22.25	48.43 ± 11.15
^RAVLT Delayed	14.20 ± 1.09	14.18 ± 1.20	13.65 ± 1.66	13.00 ± 3.35	13.94 ± 1.43
^Logical Immediate	18.15 ± 2.95	18.53 ± 2.36	15.16 ± 3.98	9.08 ± 7.43	16.81 ± 3.83
^Logical Delayed	20.06 ± 3.02	20.56 ± 2.38	16.72 ± 4.66	8.08 ± 8.62	18.56 ± 4.34
^RCFT Delayed	21.85 ± 6.69	23.37 ± 5.58	17.20 ± 7.57	14.33 ± 10.63	20.05 ± 7.41
^FCSRT Immediate	35.33 ± 4.63	35.06 ± 4.74	30.73 ± 7.28	33.00 ± 5.89	33.22 ± 6.37
^FCSRT Delayed	13.13 ± 2.00	12.84 ± 2.01	11.54 ± 2.72	13.00 ± 0.82	12.37 ± 2.46
Executive Function					
^v^TMT-B	65.70 ± 29.36	57.28 ± 18.18	89.75 ± 58.18	214.99 ± 156.15	75.28 ± 47.24
^TOP-J	17.17 ± 4.03	17.03 ± 3.97	15.37 ± 4.20	10.14 ± 5.49	16.28 ± 4.22
^v^CT2	86.04 ± 28.43	81.01 ± 29.88	107.32 ± 45.44	253.40±172.94	95.48 ± 42.46
Processing speed					
^v^CT1	44.17 ± 17.30	38.86 ± 11.66	55.04 ± 29.71	120.50 ± 111.68	48.35 ± 25.64
^v^SDMT	72.62 ± 15.65	76.59 ± 16.43	62.04 ± 31.92	22.00 ± 18.53	68.23 ± 25.57
Language Animal fluency (MoCA)	19.90 ± 4.92	19.46 ± 4.26	16.42 ± 5.10	8.23 ± 5.09	18.13 ± 5.25
Visuospatial skills					
^Block Design	37.88 ± 7.69	39.19 ± 7.04	34.54 ± 8.21	21.14 ± 10.76	36.58 ± 8.14
^RCFT Copy	33.16 ± 3.01	33.81 ± 1.92	31.57 ± 4.11	24.07 ± 12.00	32.53 ± 3.66
Attention					
^DS-Forward Span	7.16 ± 1.32	7.08 ± 1.36	6.76 ± 1.31	6.18 ± 1.17	6.96 ± 1.33
^DS-Forward Total	11.29 ± 2.46	11.10 ± 2.36	10.43 ± 2.31	8.82 ± 2.44	10.84 ± 2.41
^DS-Backward Span	5.61 ± 1.40	5.59 ± 1.23	4.81 ± 1.38	4.00 ± 1.27	5.24 ± 1.41
^DS-Backward Total	9.92 ± 2.50	10.08 ± 2.11	8.35 ± 2.33	6.64 ± 2.42	9.22 ± 2.49
**Mood, Behavioural and lifestyle Questionnaires**					
DASS					
^v^Depression†	1.48 ± 2.18	2.25 ± 2.71	2.22 ± 2.95	3.09 ± 3.86	1.98 ± 2.69
^v^Anxiety†	1.97 ± 2.17	2.42 ± 2.47	2.46 ± 2.71	4.09 ± 4.42	2.30 ± 2.52
^v^Stress†	2.95 ± 2.90	3.87 ± 3.34	3.54 ± 3.41	5.09 ± 4.95	3.42 ± 3.27
MBI-C					
^v^Total†	1.47 ± 3.01	3.08 ± 4.88	3.00 ± 5.57	9.82 ± 8.55	2.56 ± 4.84
^v^Interest†	0.29 ± 0.96	0.76 ± 1.59	0.66 ± 1.67	1.73 ± 1.85	0.57 ± 1.47
^v^Mood†	0.45 ± 1.20	0.95 ± 1.81	0.93 ± 1.94	2.00 ± 1.67	0.78 ± 1.71
^v^Control†	0.60 ± 1.30	1.20 ± 2.13	1.14 ± 2.17	3.55 ± 4.03	0.99 ± 1.97
^v^Social†	0.07 ± 0.34	0.13 ± 0.42	0.18 ± 0.57	0.55 ± 1.04	0.13 ± 0.48
^v^Beliefs†	0.05 ± 0.59	0.13 ± 0.54	0.11 ± 0.37	2.00 ± 3.32	0.11 ± 0.61
IPAQ*(high activity)	200 (52.4%)	108 (45.4%)	278 (55.8%)	1 (9.1%)	587 (52.0%)
^v^Fried†	0.25 ± 0.50	0.37 ± 0.60	0.41 ± 0.66	1.55 ± 1.13	0.36 ± 0.62
^v^PSQI†	4.58 ± 2.59	5.63 ± 3.08	5.54 ± 3.35	6.73 ± 3.58	5.26 ± 3.10
^DemQOL†	100.96±16.05	97.67 ± 12.76	96.55 ±15.63	89.90 ± 9.21	98.18 ± 15.29


Note. †=mean ± Standard Deviation, $=Frequency (percentages), ^=higher value denotes better scores, ^v^=lower value denotes better scores; Abbreviations: CN=Cognitively Normal, MCI=Mild Cognitive Impairment, SCD=Subjective Cognitive Decline, CDR=Clinical Dementia Rating, MoCA=The Montreal Cognitive Assessment, VCAT=Visual Cognitive Assessment Test, RAVLT= Rey Auditory Verbal Learning Test, RCFT=Rey Complex Figure Test, FCSRT=Free and Cued Selective Reminding Test, TMT-B= Trial Making Test B, TOP-J=Test of Practical Judgement, CT=Colour Trials, SDMT=Symbol Digit Modalities Test, DS=Digit Span, DASS=Depression Anxiety Stress Scales, MBI-C=Mild Behavioural Impairment-Checklist, IPAQ=International Physical Activity Questionnaire, Fried=Fried Phenotype Frailty, PSQI=Pittsburgh Sleep Quality Index, DemQOL=Dementia-Quality of Life Instrument

## Discussion

BIOCIS aims to investigate the progression of cognitive decline, associated neurobiological and behavioural changes in a Southeast Asian cohort as they progress along the continuum from preclinical to dementia stages. The current data highlights differences in demographics, blood biomarker analyses, neuroimaging findings as well as neuropsychological and behavioural findings between cognitively normal(CN) and impaired participants(SCD, MCI and Dementia). The impaired groups were older, had lower group means for years of education, and higher percentages of vascular risk factors(i.e., hypertension, hyperlipidaemia, diabetes).

Neuropsychological and behavioural assessments revealed a clear pattern of worsening cognition and behaviours as the group disease state progressed. Across all domains of cognition, the impaired group had lower scores than the CN group. This was reflected in the lifestyle and behavioural data as well, as more neuropsychiatric symptoms were reported in the MCI group than CN group. Interestingly, the SCD group had higher self-reported behavioural impairments compared to the MCI group, suggesting behavioural manifestations could begin and worsen before the person begins to experience cognitive decline. However, this is speculative and requires furthers investigation.

In the blood biomarker analyses, the CI groups had higher means across all biomarkers compared to the CU group. The impaired cohort had higher levels of lipids and sugars, and demonstrated a significant higher level of biomarkers (i.e., GFAP, P-tau181, Oaβ, Aβ42 and Aβ40) in the blood compared to the healthy controls. With regards to APOE genotypes, there was a slightly higher proportion of the APOE ε2 gene, in healthy control groups compared to CI. For the APOE ε4 gene, the reverse was true, with this gene being in higher proportions in the MCI and dementia group. This observation is in line with current research indicating that the APOE ε2 allele may confer some level of protection against the development of dementia, and APOE ε4 is associated with earlier onset and a higher likelihood of developing dementia (60). Our findings indicate our BIOCIS cohort has a lower prevalence of people carrying one ε4 allele compared to western cohorts, congruent with current literature. GFAP and P-tau181 could also be a potential biomarker for dementia in Southeast Asians and more research is needed.

Neuroimaging findings provided structural and functional insights into a predementia cohort. Congruent with reviewed literature, MRI scans revealed increasing white matter disease and progressive atrophy in the hippocampus as diseased group progressed. The presence and progression of WMH in Asians could reflect vascular factors, chronic inflammation, or other underlying processes contributing to cognitive decline (30). The correlation of burden of WMH in our cohort with cognitive impairment suggests vascular risk factors and VAD prevention strategies could be more relevant within the Southeas Asian population. White matter abnormalities can disrupt neural communication and exacerbate cognitive deficits, making them a crucial focus for early detection and interventions. With completion of data collection, the BIOCIS cohort could be compared with Western cohorts such as Alzheimer's Disease Neuroimaging Initiative(ADNI) and UK Biobank to study phenotypical differences between ethnicities.

Data collection is ongoing. By characterizing the neuroimaging, blood, cognitive, retinal and microbiome biomarkers in CU and impaired individuals, BIOCIS hopes to be a trial ready cohort to guide design of pharmacological and non-pharmacological interventions. With completed longitudinal data, it is anticipated that multimodal biomarkers for earlier detection of dementia will be identified. Thereafter, we intend to develop assessment protocols and risk algorithms to calculate probabilities of disease progression, design and validate digitalized assessments, as well as run interventional studies and clinical trials to investigate the prevention of dementia. By advancing our understanding of dementia in Asians with BIOCIS, we endeavour to develop effective strategies for early identification and management of cognitive impairment.

## Conclusion

The objective of this paper was to convey the protocol and study design using the preliminary data release from BIOCIS. It is not in the scope of this paper to draw major conclusions as data collection is ongoing. Over time, the systematic study and follow-up of the BIOCIS cohort will enable the identification of multifaceted pathology trajectories, deepen understanding of epidemiology of dementia, and reversible risk factors in a Southeast Asian population. Therefore, we envision the utility of the BIOCIS cohort to increase as greater sample size and length of follow up is achieved with time. Completion of BIOCIS longitudinal research aims to provide insights regarding risk-stratification of populations to guide public healthcare and precision medicine for better patient outcomes in the prevention and treatment of dementia.

## Electronic supplementary material

Supplementary Table 1. Biomarkers and Cognition Study, Singapore (BIOCIS) Study Variables

*Funding:* This project was funded by the Strategic Academic Initiative (SAI) Grant (SP1CLNT900-NTU-A630-PJ-03INP001400A630) from Lee Kong Chian School of Medicine, Nanyang Technological University, Singapore, Singapore Ministry of Education (MOE) Academic Research Fund (AcRF) Tier 3 Award MOE2017-T3-1-002 and National Medical Research Council, Singapore under its Clinician Scientist Award (MOH-CSAINV18nov-0007).

*Data availability statement:* Data is not publicly available and may be made available by the corresponding author upon reasonable request.

*Declaration of conflicting interests:* On behalf of all authors, the corresponding author states that there is no conflict of interest.

*Ethics statement:* Informed consent was obtained from all patients according to the Declaration of Helsinki and local clinical research regulations, and procedures used in the study were in accordance with ethical guidelines. This study has been approved by the NTU Institutional Review Board (NTU-IRB-2021-1036).
