# Supplementary material for: Biomarkers and Cognition Study, Singapore (BIOCIS): Protocol, Study Design, and Preliminary Findings
Source: J Prev Alzheimers Dis. 2024 May 21;11(4):1093–105. doi: 10.14283/jpad.2024.89 (PMC11266377; doi:10.14283/jpad.2024.89)
Supplement: Supplementary file 1 — Supplementary Table 1. Biomarkers and Cognition Study, Singapore (BIOCIS) Study Variables [file mmc1.docx]

Supplementary Table 1. Biomarkers and Cognition Study, Singapore (BIOCIS) Study Variables

|  | **Timepoints** | **Assessment** | **Range** |
| --- | --- | --- | --- |
| ***Cognitive Assessments*** | 1, 2, 3, 4, 5 | Montreal Cognitive Assessment (MoCA) | 0-30 |
|  |  | Visual Cognitive Assessment Test (VCAT) | 0-30 |
|  |  | Clinical Dementia Rating (CDR) Scale | 0-3 |
|  |  | Rey Auditory Verbal Learning Test (RAVLT) | Learning = 0-75  Delayed = 0-15  Recognition = 0-15 |
|  |  | Rey–Osterrieth Complex Figure (ROCF) | Copy = 0-36, Time taken (secs)  Immediate = 0-36, Time taken (secs)  Delayed = 0-36, Time taken (secs) |
|  |  | Free and Cued Selective Reminding Test (FCSRT) | Immediate Recall Free Trial = 0-16  Immediate Recall Cued Trial = 0-16  Delayed Recall Free Trail = 0-16  Delayed Recall Cued Trail = 0-16 |
|  |  | Test of Practical Judgment (TOP-J) | 0-45 |
|  |  | Color Trails Test 1 (CTT-1) | Time taken (secs) |
|  |  | Color Trails Test 2 (CTT-2) | Time taken (secs) |
|  |  | Trail Making Test B (TMT-B) | Time taken (secs) |
|  |  | WMS Logical Memory | Immediate = Average of 2 trials, 0-25  Delayed = 0-25 |
|  |  | WAIS Digit Span | Forward = 0-16  Backward = 0-16 |
|  |  | WAIS Block Design | No time bonus = 0-48  Time bonus = 0-66 |
|  |  | Symbol Digit Modalities Test (SDMT) | 0-135 |
| ***Mood, Behavioural, and Lifestyle Questionnaires*** | 1, 2, 3, 4, 5 | Depression Anxiety Stress Scales (DASS) | Depression = 0-42  Anxiety = 0-42  Stress = 0-42  Total = 0-126 |
|  |  | Mild Behavioural Impairment-Checklist (MBI-C) | Interest = 0-18  Mood = 0-18  Control = 0-36  Social = 0-15  Beliefs = 0-15  Total = 0-102 |
|  |  | International Physical Activity Questionnaire (IPAQ) | 1-3 |
|  |  | Fried Phenotype Frailty  Walking speed (4m)  Hand grip strength | 0-5  Time taken (secs)  Isometric grip force (kilograms) |
|  |  | Pittsburgh Sleep Quality Index (PSQI) | 0-21 |
|  |  | Dementia-Quality of Life Instrument (DemQOL) | 0-116 |
|  |  | Subjective Memory Complaint Questionnaire (SMCQ) | 0-14 |
| ***Neuroimaging Visual Ratings*** | 1, 2, 3 | Modified Fazekas Scale | 0-12 |
|  |  | Perivascular spaces (PVS) Grade | 0-4 |
|  |  | Staals’ Criteria | 0-4 |
|  |  | Scheltens’ Medial temporal atrophy (MTA) Score | 0-4 |
|  |  | Cerebral Microbleeds | Visual counting of number in bilateral basal ganglia and lobar brain regions |
|  |  | Lacunae | Visual counting of number in frontal and non-frontal regions in both hemispheres |
| ***Blood Biochemical Test*** | 1 | Total cholesterol (TC) | mmol/L |
|  |  | High-density lipoprotein cholesterol (HDL-C) | mmol/L |
|  |  | Low-density lipoprotein cholesterol (LDL-C) | mmol/L |
|  |  | Glycated hemoglobin (HbA1c) | % |
| ***Plasma Biomarkers*** | 1, 2, 3 | Apolipoprotein E (APOE) | Genotype (E2/E2, E2/E3, E3/E3, E3/E4, E4/E4, E2/E4) |
|  |  | Aβ oligomers (Oaβ) | ng/ml |
|  |  | Neurofilament Light Chain (NFL) | pg/mL |
|  |  | Amyloid Beta 42 (Aβ42) | pg/mL |
|  |  | Amyloid Beta 40 (Aβ40) | pg/mL |
|  |  | Glial fibrillary acidic protein (GFAP) | pg/mL |
|  |  | Phosphorylated Tau at position 181 (pTau-181) | pg/mL |
| ***Retinal scans*** | 1, 2, 3 | Under definition and deliberation | |
| ***Microbiome*** | 1, 2 | Under definition and deliberation | |
